# Strengthening the Informed Consent Process in International Health Research through Community Engagement: The KEMRI-Wellcome Trust Research Programme Experience

**DOI:** 10.1371/journal.pmed.1001089

**Published:** 2011-09-13

**Authors:** Mwanamvua Boga, Alun Davies, Dorcas Kamuya, Samson M. Kinyanjui, Ester Kivaya, Francis Kombe, Trudie Lang, Vicki Marsh, Bibi Mbete, Albert Mlamba, Sassy Molyneux, Stephen Mulupi, Salim Mwalukore

**Affiliations:** 1 Consent and Communication Committee, KEMRI-Wellcome Trust Research Programme, Kilifi, Kenya; 2 Nuffield Department of Clinical Medicine, Oxford University, Oxford, United Kingdom

## Abstract

Samson Muchina Kinyanjui and colleagues from the KEMRI-Wellcome Trust Research Programme discuss how they modified their informed consent processes by taking into account local social, cultural, and economic contexts in the design and administration of consent forms.

**Summary Points**

- Informed consent is fundamental to ethical health research.
- Significant challenges are experienced in obtaining consent for research, particularly in poor settings.
- Consenting processes can be strengthened by taking into account local social, cultural, and economic contexts in the design and administration of consent forms.
- Institutional wide support is important in ensuring consistency in the consenting process for all studies within a given institution.

## The Challenge

Informed consent is fundamental to ethical health research. However, significant challenges are experienced worldwide in ensuring regulatory and practical requirements for informed consent are met [1]–[4]. These challenges are partly attributable to differences in the understanding of research concepts and processes between researchers and research participants, differences that are most acute where there are large gaps between these groups in access to resources, literacy levels, and perceptions of health and illness, and in contexts where access to biomedical health care is severely constrained [1],[5]. This paper describes a programmatic approach to strengthening consent processes in a low resource setting and aims to contribute to global dialogue on practical ways of strengthening informed consent processes for health research.

The Kenya Medical Research Institute (KEMRI)-Wellcome Trust Research Programme (KWTRP) in Kilifi, Kenya, is a collaborative multidisciplinary research programme established in 1979 that focuses on the major causes of ill health in Kenya and sub-Saharan Africa. The Programme faces many of the challenges mentioned above. Studies in the area on community perceptions [1],[6]–[9] and institutional experiences of community engagement in research [10],[11] have highlighted a number of issues that undermine informed consent processes, including:

- Limited exposure through formal education to research concepts and procedures, and lack of local terms for key elements of research.
- Rumours about research activities and their purpose, for example on reasons for taking blood samples from well children.
- Difficulties for potential participants or their guardians in listening to or understanding large volumes of research-related information, especially when children are sick and consenting processes are considered to be delaying initiation of treatment.
- Perceptions that research procedures are part of standard care (therapeutic misconceptions) [12],[13], or vice versa [14].
- Lack of in-depth understanding of research or research ethics among those responsible for explaining research activities.

## Development of Contextualized Informed Consent Form Templates and Consenting Procedures

KWTRP has a large number of studies going on concurrently, and ensuring research is conducted to the highest scientific and ethical standards is a key Programme goal. To address the challenges identified in relation to consent processes, and to ensure harmonization and careful review of consenting processes, a committee designated “Consent and Communication Committee” (CCC) was constituted in 2005. Members of the CCC are drawn from different departments within the Programme, including laboratory-based staff, clinicians, social scientists, nurses, clinical trial coordinators, and community facilitators. The committee meets on a monthly basis to review consent forms and processes for all new proposed studies.

The CCC members were concerned that many consent forms were unnecessarily long, complicated, and, importantly, failed to take into account local priorities and concerns identified through the research and activities described above, while at times missing key elements required nationally or internationally [15]–[17]. In an effort to improve the situation, the committee drew upon internationally available templates [18]–[20] and on local research and insights to develop locally contextualized templates for each of the four main types of research conducted by the Programme: 1) clinical trials; 2) sampling only involving no intervention; 3) observational studies involving no sampling or interventions; and 4) interview only studies. Some issues and concerns might be more relevant to one type of study than another. For example, interventions and sampling studies might raise higher safety concerns than an interview only study, while the latter might raise more salient confidentiality issues as it is harder to disguise a recorded voice than it is to anonymise a sample. Table 1 highlights the key communication issues impacting consent processes in our context, and how these were tackled in the consent templates. Wording of key elements of the consent templates has been tested through ongoing community engagement activities.

**Table 1:** Key communication issues addressed in contextualised ICF templates.

Common Communication Issues	How Addressed in ICF Design
Individually developed ICFs that miss vital information, inconsistency between ICFs from similar studies, lack of input from social science research, poor translation.	Formation of a centralised consenting support and review structure.Development of contextualized templates for different categories of studies and their translations.
Failure to take context adequately into account, e.g., participants' health worries.	Requirement of an SOP for the consenting process that takes account of context.
Length and structure of ICF undermines understanding key messages of study, particularly among the poorly literate who often form the majority of participants.	CCC reviews each new ICF and suggests ways of keeping it short and simple while fulfilling all the regulatory requirements. For example, complexity is often reduced by presenting multiple procedures in a bulleted list rather than in continuous prose.
Difficulty in differentiating between research interventions and standard care (therapeutic misconceptions).Confusion between routine diagnostic samples and research samples with linked expectation that results will always be returned because of their assumed implications for the health of the individual providing sample.	ICF begins with brief information about nature of research in general, the research institution, and its relationship to the District Hospital, followed by brief explanation of health condition affecting the participant, and the standard care for that condition, before delving into details of the proposed study.A statement on whether or not the study result will have direct implication on decisions for care of current illness and or the participants' health in general is inserted into the ICF.
Difficulty in communicating unfamiliar research concepts, e.g., randomization and controls, placebo, standard versus test intervention.	Development of simpler terminology through discussions with researchers, field staff, and community liaison group. In addition, supplementary information for clarification where needed is provided alongside the ICF.Examples of terminology simplification: Randomisation is a difficult concept to translate, since most translations of “chance” also imply “luck”, and are considered non-neutral. Eventually the phrase *“ a sytem such that everyone has the same chance of being included in the study, without favouritism”* was adopted. Confidentiality: Kiswahili word “*siri*” or “*secret*” had been in common use, but found to have negative connotations in community as well as being inaccurate. Instead the phrase “*a limited number of people closely concerned with the research*”, was adopted. Standard versus test intervention—“standard” is explained as the *“ treatment recommended by the Ministry of Health”*.
Confusion between reimbursements/compensations and benefits. In poor settings, even bus fare given for follow-up or surveillance visits could be seen as a benefit since it is possible to make a saving on it, e.g., by walking part of the journey.	Compensation/reimbursements included under risks/costs, rather than under benefits, to emphasise their nature, e.g., “*You will be asked to come back to the clinic after one month, which will take your time, but the costs of travelling will be refunded*”.
Difficulty in understanding the level of risk—e.g., how much blood can be withdrawn safely, particularly from an already sick child.Anxiety over adverse events and what would happen if they occur.	Find simpler ways of explaining risk, e.g., for blood samples, explaining sample volumes in locally understandable terms (teaspoons, bottle tops) as well as milliliters.Clear explanation on what safety monitoring structures are in place, e.g., “*Your child will be continuously monitored by a doctor who may withdraw her from the study in case of any adverse events*”.
Concerns over what will happen to samples, particularly with respect to storage and exportation.	Explicit disclosure of all planned exportation and storage with separate consent being requested for each action.“*A part of the sample will sent to labs in Oxford university in the UK for test x*”.Statement on the fact the any future research on stored sample must first be approved by national ERC.

Most informed consent processes in Kilifi take place in a local language, usually Kiswahili or Kigiriama. However, informed consent forms (ICFs) are usually developed in English by researchers as part of the proposal development process, and later translated into local languages. The CCC was aware of major limitations of many translations of consent forms and so they adopted a systematic approach to translation of ICF templates to Kiswahili versions. A series of workshops involving community facilitators (who are native speakers of the local languages and Kiswahili), nurses, scientists, and a professional translator (all competent Kiswahili speakers) were held in which each concept covered in the templates was written directly in the local languages, rather than simply translated from the English. The resulting Kiswahili templates were checked for accuracy and meaning by community facilitators not involved in the initial development.

The design of an ICF is only part of a wider consent process [21]. Thus, in addition to enhancing the ICF design, the CCC designed a template for developing a standard operating procedure (SOP) to guide researchers to consider key elements of the wider consenting process for their studies. These elements include training of those who will administer the ICF, considering the implications and flexibility of the timing of the consenting process (for example, whether it will happen before, during, or after admission of patient to the ward), and considering possibilities for re-visiting information over time. The SOP also prompts researchers to consider what supportive information about the illness or about the context might be important for staff conducting the informed consent process to answer participants' questions.

## Experience of Using Contextualized ICF Templates and SOP in Kilifi

Box 1 summarises the output of the consent strengthening process. The contextualised ICF templates have been in use for over two years now and were used in the majority of approximately 90 new research proposals that covered all types of studies developed between May 2008 and November 2009. Although more research is needed to systematically assess the impact of this enhanced consent process (including contextualized ICFs), informal responses concerning this programmatic approach to strengthening consenting include:

- Appreciation of the support that this approach provides for developing consent processes: Practically all researchers now engage the CCC for input in their studies' ICF design and in discussions around any consenting issues anticipated for their studies.
- A perception among CCC members that availability of contextualised templates and their corresponding translations have simplified ICF development, and contributed to relatively clear and consistent ICFs that are relevant to the local context.
- Researchers who have developed study-specific consent SOPs report that they have been a useful tool in training study staff, and in providing an ongoing reference over the course of their studies.
- The two main ethics committees that review research done in KWTRP, the Kenya National Ethics Review Committee (ERC) and the Oxford Tropical Medicine Ethical Research Committee (OXTREC), have responded supportively to the templates. The Kenya ERC will be making a formal review of these forms with the aim of making these available to other researchers in Kenya. OXTREC welcomed the templates as a practical guidance tool and have requested that these are made available as examples to other researchers.
- The Global Health Trials Web site (http://ght.globalhealthehub.org/), a Web-based international support platform, has uploaded the templates as part of responding to many requests for examples of consent forms for different types of research.

**Box 1. Summary of the Output of the Consent Strengthening Process**

1. A committee that provides systematic support for ICF design and for development of consent processes.
2. A set of standardized, locally specific templates for ICFs in Kiswahili and English that cover the types of studies commonly undertaken, and which draw on social science research and community engagement processes within the Programme to address common communication issues.
3. SOP guidelines that prompt researchers to consider the wider context within which informed consent forms will be used, including training and monitoring of consent administrators.

## Challenges for Developing a Locally Relevant ICF and Consenting Process

An important challenge for the development of locally specific ICF templates concerns generating and maintaining sufficient understanding of relevant local community and research contexts. This requires developing good community engagement structures and capacity to conduct good quality health research. Similarly, developing locally relevant research terms and their corresponding translations requires a good understanding of research and the ability to draw on a wide range of expertise in different research areas. For example, to address the risks of conflation between research and treatment amongst patients, research participants, and staff in Kilifi, the CCC drew on clinical researchers' expertise to develop an agreed definition of local “standard of care”.

As acknowledged for research ethics review processes in general [22], the work of the CCC relies on a range of conditions, including staff expertise, commitment, and availability alongside normal responsibilities. Although the use of templates has facilitated review processes, there are also communication challenges for an internal review group in supporting their colleagues to develop informed consent processes. For example, SOP templates, which are a crucial component of the strengthened consent process, have not been applied as consistently as have the consent templates. This may be because currently consent SOPs are not included in the wider proposal review process undertaken before any study can begin. The CCC is therefore keen to ensure that SOPs are reviewed in advance. The challenge will be to do this in a way that is seen as supportive of researchers' efforts to oversee strong consent processes, rather than a new bureaucratic hurdle.

The adaptation of the templates cited here in other settings is likely to require similar steps for context-specific development relevant to participant communities and research staff, types, and institutions. Experiences in Kilifi suggest that locally adapted communication processes that combine the development of contextualised ICF templates and ongoing supportive processes for their use are a valuable investment of resources through their potential to strengthen informed consent, particularly in international research. The ICF and SOP ICF templates described in this paper are available on the Programme's Web site (http://www.kemri-wellcome.org/) and the Global Health Trials Web site (http://ght.globalhealthehub.org/).

## Abbreviations

CCC: Consent and Communication Committee
KEMRI: Kenya Medical Research Institute
KWTRP: KEMRI-Wellcome Trust Research Programme
SOP: standard operating procedure
